# Cobalt Oxide Porous Nanofibers Directly Grown on Conductive Substrate as a Binder/Additive-Free Lithium-Ion Battery Anode with High Capacity

**DOI:** 10.1186/s11671-017-2058-0

**Published:** 2017-04-26

**Authors:** Hao Liu, Zheng Zheng, Bochao Chen, Libing Liao, Xina Wang

**Affiliations:** 1 School of Science, China University of Geosciences, Beijing, 100083 People’s Republic of China; 2 Hubei Collaborative Innovation Center for Advanced Organic Chemical Materials, Hubei Key Laboratory of Ferro and Piezoelectric Materials and Devices, Faculty of Physics and Electronic Science, Hubei University, Wuhan, 430062 People’s Republic of China; 3 School of Materials Science and Technology, China University of Geosciences, Beijing, 100083 People’s Republic of China; 4 Hubei Key Laboratory of Ferro and Piezoelectric Materials and Devices, Faculty of Physics and Electronic Science, Hubei University, Wuhan, 430062 People’s Republic of China; 5 Beijing Key Laboratory of Optical Detection Technology for Oil and Gas, China University of Petroleum, Beijing, 102249 People’s Republic of China

**Keywords:** Cobalt monoxide, Nanofibers, Anode, Li-ion battery, Binder free

## Abstract

In order to reduce the amount of inactive materials, such as binders and carbon additives in battery electrode, porous cobalt monoxide nanofibers were directly grown on conductive substrate as a binder/additive-free lithium-ion battery anode. This electrode exhibited very high specific discharging/charging capacities at various rates and good cycling stability. It was promising as high capacity anode materials for lithium-ion battery.

## Background

Transition metal oxides usually exhibit remarkably high specific capacities as lithium-ion battery anode materials, and they are believed to be promising anode materials to replace the commercial graphite anode [1–5]. Among various transition metal oxides, cobalt monoxide (CoO) has drawn great attention due to its high theoretical capacity of 716 mA h g^−1^ which is nearly twice than that of graphite anode [2, 6]. Nevertheless, this anode material suffers from large volumetric change during discharging/charging cycles, leading to pulverization and capacity fading of bulk electrode. Nanostructural design with specific architectures has been demonstrated as an effective way to alleviate the volumetric change of active materials, such as Si [7], Ge [8], and transition metal oxides [6, 9], because lots of voids or pores’ presence in the nanostructures can accommodate the volumetric expansion of the active materials during lithiation process. Specially, one-dimensional (1D) nanostructures such as nanowires or nanofibers are proved to be an optimized architecture for the electrochemical electrodes due to their well-defined charge transport path, high specific surface area, and high porosity features [6, 10–12]. Nevertheless, in traditional battery electrode preparation, these 1D nanostructures are usually mixed with binders and conductive additives before being tape casted on a flat current collector to enhance mechanical and electrical properties of electrodes [12, 13]. The introduction of binders and additives not only reduces the energy density of the electrode but also generates undesired interfaces between the active materials and the additives, which increases the complexity of charge transfer process [14].

In the present work, CoO porous nanofibers were directly grown on conductive substrate as a binder/additive-free anode for lithium-ion battery. These porous nanofibers exhibited high specific discharging/charging capacities at various rates and good cycling stability, suggesting its promising application as lithium-ion battery anode.

## Methods

### Fabrication of CoO Nanofibers on Conductive Substrate

Typically, 1 mmol cobalt dichloride, 5 mmol urea, and 10 mmol sodium chloride were dissolved in 30 mL of distilled water under vigorous stirring for 20 min to form homogeneous pink solution. The previous solution was transferred into a Teflon-lined stainless steel autoclave, and then a 2 cm × 2 cm titanium foil which had been cleaned by dilute hydrochloric acid was put into the autoclave, followed by heating for 10 h at the temperature of 100 °C. The products with titanium substrate were taken out, washed with distilled water, and then dried at 60 °C for 1 h. Finally, the as-prepared products were annealed at the temperature of 500 °C for 2 h under an N_2_ atmosphere.

### Characterization

The crystallinity of the products was examined using an X-ray diffraction (XRD) with Cu-Kα radiation (Bruker D8). The morphologies and compositions of products were characterized by a field-emission scanning electron microscope (SEM; JSM-7100F) and a transmission electron microscope (TEM; JOEL 2100) equipped with an energy-dispersive X-ray (EDX) spectrometer.

### Electrochemical Properties of the CoO Nanofiber Anode

The electrochemical properties of the samples were examined using CR2032 coin-type cells with Li foil as counter electrode. No binder or conducting carbon was used during the cell assembly. The liquid electrolyte was 1.0 M LiPF_6_ in ethylene carbonate/diethyl carbonate solvent (Shenzhen Kejing Star Technology C., Ltd.). All coin cells were cycled between 0.01 and 3.0 V at different rates on a battery test system (CT4008W, Neware Co., Ltd.). The electrochemical impedance spectroscopy (EIS) of the batteries was collected in the frequency range from 100 kHz to 0.5 Hz under an alternating current (AC) stimulus with a 10 mV of amplitude (Vertex, Ivium Electrochemical Measurement System). After the cycling test, the coin cells were disassembled to characterize the morphologies change of the electrode.

## Results and Discussion

**Fig. 1:**
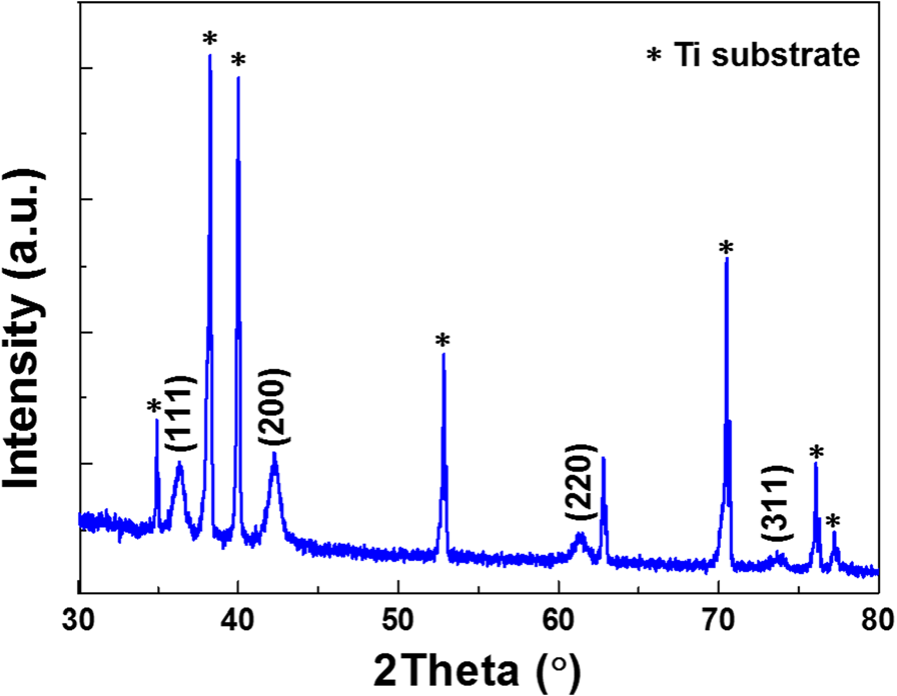
XRD spectrum taken from the CoO sample grown on Ti substrate

Figure 1 shows XRD peaks of the prepared sample, which can be indexed as (111), (200), (220), and (311) of cubic CoO (JCPDS No. 48–1719). The diffraction peaks of Ti come from the substrate. In addition, the grain size of CoO sample is estimated as ~11 nm using the Scherrer equation.

**Fig. 2:**
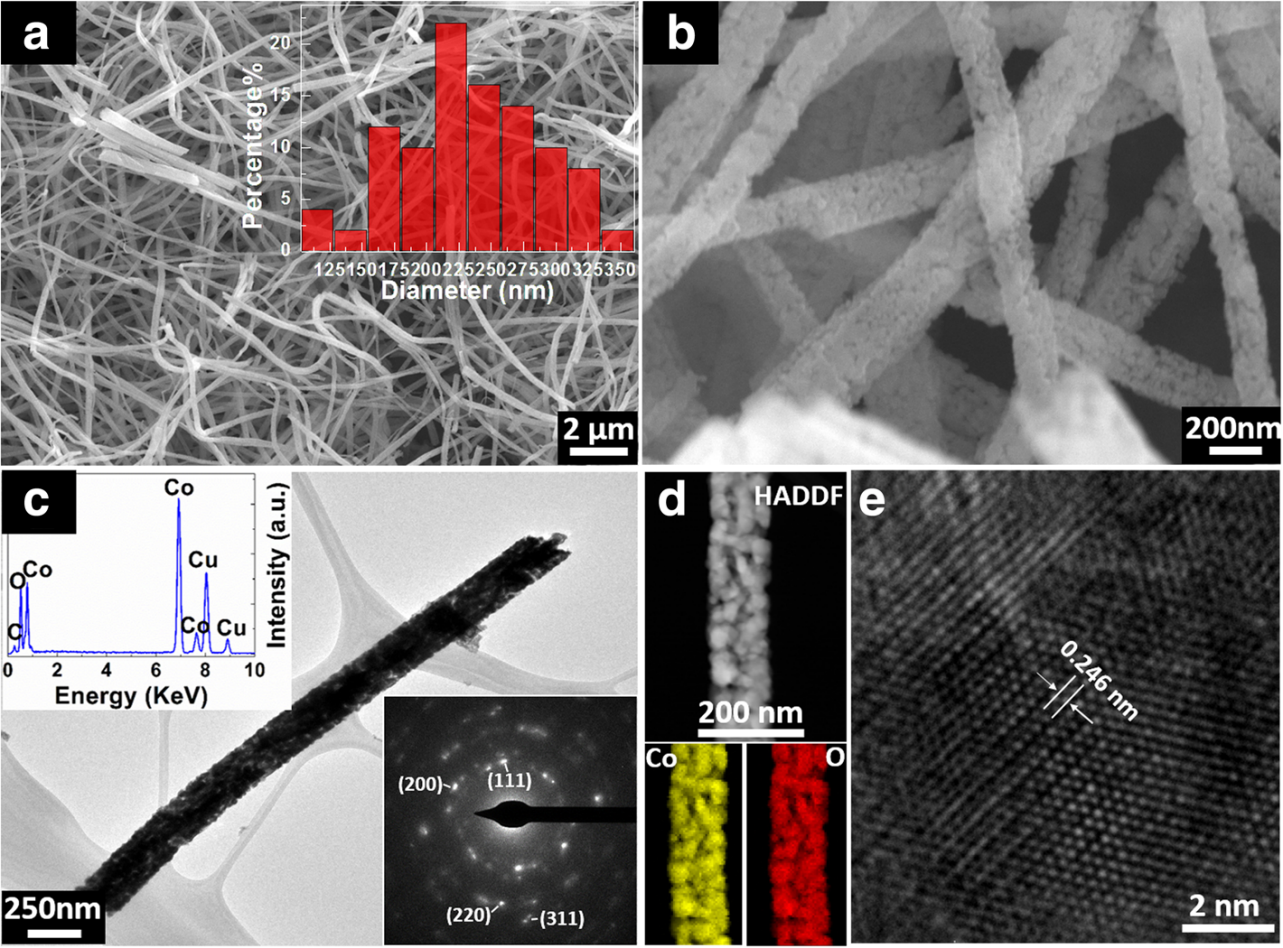
**a** Low-magnified and **b** high-magnified SEM images of CoO nanofibers. Size distribution of the nanofiber diameters is shown in *inset* of **a**. **c** Low-magnified TEM images of a typical single CoO nanofiber. EDX spectrum and electron diffraction patterns are shown in **c**, respectively. **d** High-angle annular dark field (HAADF) image taken from part of this nanofiber. Co and O EDX elemental maps taken from the sample region are also shown in **d**. **e** High-magnified TEM (HRTEM) image taken from the nanofiber

In order to reveal the morphologies of the prepared CoO sample, SEM was carried out, which results are shown in Fig. 2a, b. Many ultralong nanofibers with diameter of ~103–360 nm (average diameter ~236 nm) are woven together (Fig. 2a). Furthermore, high-magnified SEM image discloses these long nanofibers that are composed of lots of nanoparticles (Fig. 2b). This characteristic is further confirmed using TEM technique. We have observed many nanofibers using TEM, and a typical one is shown in Fig. 2c, d. It is clearly shown that the nanofiber contains many nanoparticles. The average size is estimated as ~12 nm, being consistent with previous XRD result. In addition, the nanofibers are composed of Co and O elements (Cu signals come from Cu grid for TEM observation), revealing by EDX (upper left inset of Fig. 2c) and elemental mapping results (Fig. 2d). Moreover, the selection area diffraction pattern (SAED) taken from the nanofiber (bottom right inset of Fig. 2c) shows the diffraction rings that can be indexed as (111), (200), (220), and (311) of cubic CoO, being consistent with XRD results. High-resolution TEM (HRTEM) image (Fig. 2e) reveals the lattice structure of the nanofibers. The measured d-spacing of the lattices is ~0.246 nm, corresponding to the (111) plane of cubic CoO.

The formation mechanism of the porous CoO fiber can be explained as the following [15]. In the hydrothermal process, the hydrolysis-precipitation process of urea took place and numbers of CO_3_^2−^ and OH^−^ anions were gradually produced. Then, cobalt-hyroxide-carbonate nucleus (Co(OH)_x_(CO_3_)_0.5(2−x)_·nH_2_O) were formed and continuously grew along specific orientation preferentially on the metal substrate. As a result, cobalt-hyroxide-carbonate nanowires were obtained. During the next annealing process under the Ar gas protection, the as-prepared nanowires decomposed to release CO_2_ and H_2_O and, then, porous CoO nanofibers were obtained.

**Fig. 3:**
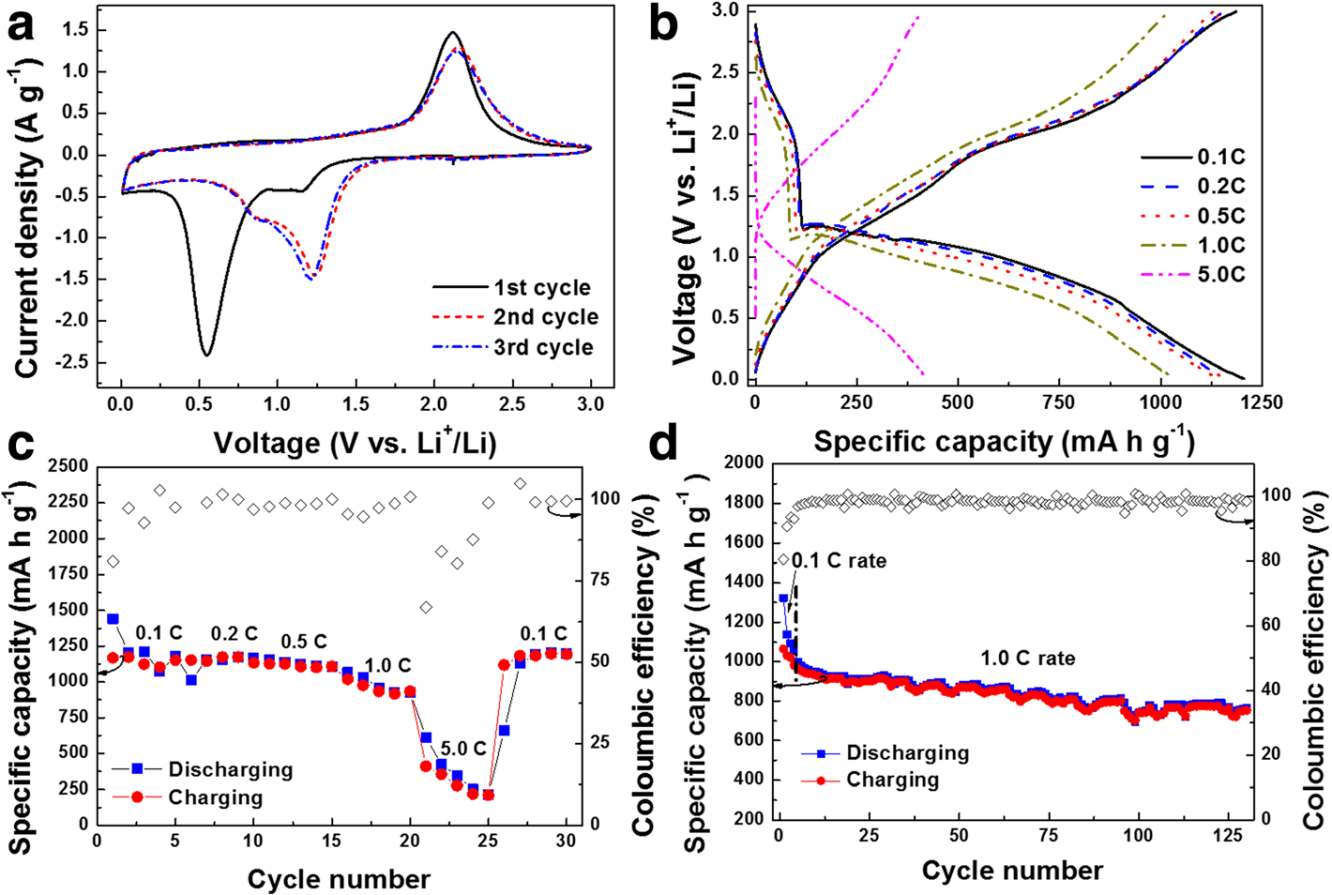
**a** CV plots of the CoO anode at scan rate of 0.5 mV s^−1^ in the voltage range of 0.0–3.0 V vs. Li^+^/Li during the first three cycles. **b** Discharging/charging voltage profiles of the CoO anode at various rates in the voltage range of 0.0–3.0 V vs. Li^+^/Li (these plotted profiles obtained from the second cycle at each rate). **c** Discharging/charging capacities of the CoO anode cycled at different rates. **d** Cycling characteristic of this electrode at 1.0 C rate. Their corresponding coulombic efficiencies are also shown in **c** and **d**

Figure 3a shows cyclic voltammogram (CV) of the CoO anode at scan rate of 0.5 mV/s in voltage range of 0.0~3.0 V vs. Li^+^/Li. In the first cathodic scan, a wide hump between ~1.4–0.9 V vs*.* Li^+^/Li is firstly observed, coming from the decomposition of electrolyte and the formation of solid-electrolyte interlayer (SEI) on the electrode surface [15]. Following the wide hump, a reduction peak locates at ~0.55 V vs. Li^+^/Li, which can be attributed to the phase transition from CoO to Co and Li_2_O [3]. Nevertheless, the corresponding reduction peak shifts to a higher voltage of ~1.23 V vs. Li^+^/Li during the next cycle, probably because of the pulverization of the CoO nanoparticles during the discharging/charging processes [16]. On the other hand, one peak is found at ~2.12 V vs. Li^+^/Li during the first anodic scan, indicating the oxidation of Co^0^ to Co^2+^ [2]. In the subsequent cycle, the anodic peak slightly shifts to high voltage as ~2.14 V vs. Li^+^/Li. In addition, the CV curves obtained from the 2nd and 3rd cycles are almost the same, indicating good cyclability of the CoO electrode.

Figure 3b shows the discharging/charging voltage profiles taken from the second cycle at various rates in the voltage range of 0.0–3.0 V vs. Li^+^/Li. The lithiation voltage shows a sloping profile below 1.25 V vs*.* Li^+^/Li, being consistent with the CV results. In addition, the CoO anode exhibits high discharging/charging capacities at different rates, such as 1205/1186, 1158/1156, 1141/1130, 1033/1021, and 423/410 mA h g^−1^ at rates of 0.1, 0.2, 0.5, 1.0, and 5.0 C. It is noticed that the specific capacities of the CoO anode at various rates are significantly higher than its theoretical capacity of 716 mA h g^−1^. Large additional capacities were always observed in transition metal oxide anode materials, which are probably contributed by pseudocapacitance of nano-sized materials [2] or the formation of oxygen-rich transition metal oxide materials [17]. Furthermore, this electrode also shows good capacity retention, as its discharging/charging capacities can be recovered to ~1202/1196 mA h g^−1^ when the rate returns to 5.0 from 0.1 C rate (Fig. 3c). It is worth to note that the initial coulombic efficiency is ~81%, much higher than those in other reports [2, 18]. In addition, the coloumb efficiency remarkably decreases when the charging/discharging current density increased from 1 to 5 C rate. This may result from the instability of the electrode during the drastic change of current, e.g., new SEI layer formed on the electrode surface. After four charging/discharging cycles, the coloumbic efficiency increases to 99%, indicating the stabilization of the electrode. The cycling performance of the electrode is shown in Fig. 3d. A capacity of ~981 mA h g^−1^ is obtained at 1.0 C rate, and a slow decay to ~764 mA h g^−1^ is found after 130 cycles. This is to say, only 0.17% of the initial capacity is lost in each cycle. Moreover, the coulombic efficiency remains above 99% during the cycling test.

**Fig. 4:**
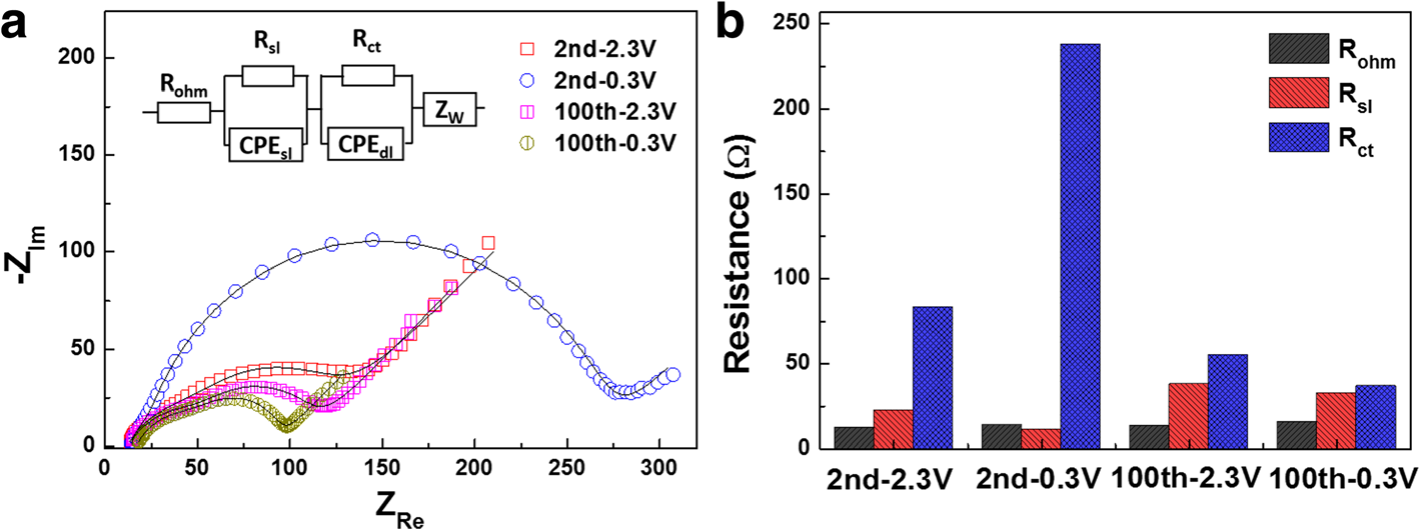
**a** Nyquist plots of the CoO anode at voltage of 2.3 and 0.3 V vs. Li^+^/Li during the 2nd and the 100th discharging cycles. The *inset* shows the equivalent circuit model for this electrode. *R*_ohm_ stands for series ohmic resistance. *R*_sl_ and CPE_sl_ represent surface layer resistance and capacitance, respectively. *R*_ct_ and CPE_dl_ represent the charge transfer resistance and double layer capacitance. *Z*_w_ represents Warburg impedance. The *lines* in these figures are the fitted curves using this model. **b** Comparison of the individual real impedance, *R*_ohm_, *R*_sl_, and *R*_ct_ obtained from the Nyquist plots

In order to further understand the cycling process of the CoO anode, EIS measurements were carried out at the voltage of 2.3 and 0.3 V vs. Li^+^/Li during the 2nd and the 100th discharging cycles. Figure 4a gives the corresponding Nyquist plots. In general, a typical Nyquist plot consists of three characteristic features: two depressed semicircles in the high and medium frequency range, followed by an inclined line in the low frequency region. The first depressed semicircle at high frequency responds to the resistance of the surface-passivating layer, i.e.*,* SEI layer. The second depressed semicircle at medium frequency is related to the resistance of the charge transfer on the electrode/electrolyte interfaces. The inclined line at low frequency represents the diffusion of Li ion in the electrode, which is also named Warburg impedance. An appropriate equivalent circuit model (inset of Fig. 4a) was established to simulate the Nyquist curves, and the electrical parameters R_ohm_, R_sl_, and R_ct_ in this model can be obtained, as shown in Fig. 4b. The ohmic resistances of the CoO anode are quite stable during the cycling. Nevertheless, the resistance of SEI layer increases after one hundred cycles, indicating the SEI layer became thick during the cycling. In addition, the charge transfer resistance sharply increase when this electrode discharges from 2.3 to 0.3 V vs*.* Li^+^/Li, probably resulting from the formation of insulating Li_2_O particles in the electrode [19]. After 100 discharging/charging cycles, both charge transfer resistances obtained at 2.3 and 0.3 V vs. Li^+^/Li during the discharging fall greatly as compared to these obtained in the second discharging process. This is probably due to the surface of active material increases during the pulverization of the electrode in the cycling process, which benefits to charge transfer on the electrode/electrolyte interfaces.

**Fig. 5:**
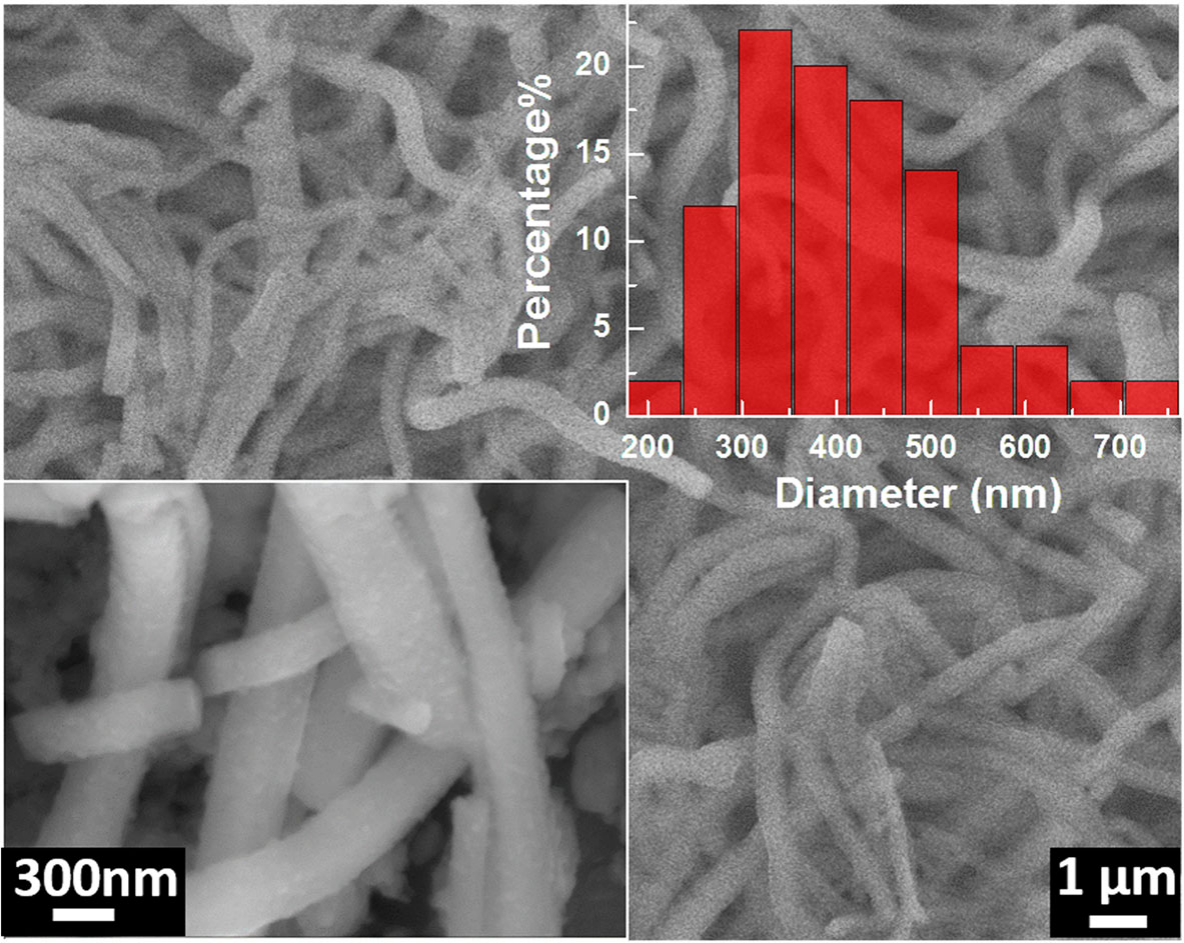
SEM image taken from the CoO anode after 130 cycling test. An enlarged SEM image and size distribution of the nanofiber diameters are shown in *inset* of Fig. 2a

In order to reveal the structural stability of this binder-free CoO anode during the cycling, the electrode after the 130 cycling test was delithiated at voltage of 3.0 V vs. Li^+^/Li and its morphology was monitored using SEM, which result is shown in Fig. 5. It reveals that the nanofibers are well preserved and adhere to the substrate during the cycling. Nevertheless, the average diameter of the nanofibers is ~409 nm which is much larger than that of the pristine CoO sample (~236 nm). This diameter increase has been observed in other transition metal oxides materials [20], which may come from the presence of the SEI layer on the surface of nanofibers and partially irreversible volumetric changes of delithiated nanofibers as compared to the pristine one [20]. On the other hand, the lots of voids between the nanofibers can buffer the volume swings, which benefits to the structural stability and cycling performance.

## Conclusions

In summary, CoO porous nanofibers, being composed of lots of nanoparticles, were directly synthesized on conductive substrate and applied as bind-free Li-ion battery anode. This anode showed very high discharging/charging capacities at various rates, such as 1205/1186 and 1033/1021 mA h g^−1^ at rates of 0.1 and 1.0 C, respectively. In addition, the porous nanofiber structure was well maintained during the cycling, leading to good cycling stability of this CoO anode. Therefore, the CoO porous nanofibers grown on conductive substrate showed promise as bind/additive-free lithium-ion battery anode.
